# Laboratory Validation of a Novel Indigenously Developed Bite Force Measuring Device

**DOI:** 10.7759/cureus.60880

**Published:** 2024-05-22

**Authors:** Madhu Ranjan, Surender Kumar, Bishnupati Singh, Amit V Mahuli, Awanindra K Jha, Shantala R Naik

**Affiliations:** 1 Prosthodontics, Dental Institute, Rajendra Institute of Medical Sciences, Ranchi, IND; 2 Public Health Dentistry and Preventive Dentistry, Dental Institute, Rajendra Institute of Medical Sciences, Ranchi, IND; 3 Orthodontics, Dental Institute, Rajendra Institute of Medical Sciences, Ranchi, IND; 4 Oral Medicine and Radiology, Dental Institute, Rajendra Institute of Medical Sciences, Ranchi, IND

**Keywords:** maximum voluntary bite force, calibration report, laboratory validation, bite force, byte

## Abstract

Introduction: It is critical to measure the maximum voluntary bite force of patients receiving restorative dentistry. A new device known as "BYTE" has been developed indigenously to measure bite force in humans. The purpose of this study is to evaluate the BYTE device's consistency and accuracy in a lab setting.

Methodology: Testing and calibration were done in the laboratory. The calibration machine with load cell pressed the biting part of the device with various forces from 3 N to 444 N in 3 N increments for two to three seconds each. The recorded force value in Newton by the device was noted down.

Results: At numerous standard loads, the minimum accuracy error is 0.333 N, while the maximum is 1.667 N. It marginally underestimates the load with an average accuracy error of 0.833 N.

Conclusion: The calibration report showed that the BYTE device is precise and reliable and can be used to measure maximum bite force.

## Introduction

The bite force is one aspect of mastication that researchers have studied to learn more about the masticatory system’s function [1]. The bite force is the force the chewing muscles use to close the teeth together. The maximum voluntary bite force (MVBF) shows how well the mouth and jaw system works. It depends on how strong and well-coordinated the jaw muscles are and how they work with the jaw bones [2,3]. In the molar region, healthy adults' natural teeth can bite with a maximum force of 300 to 600 Newtons (N). Several anatomical and physiological factors influence MVBF. Dentists use bite force to check how well different dental treatments work. They also use it to study how problems and diseases affect the chewing system, such as temporomandibular joint disorder [4].

There are many devices available globally that can measure maximum bite force (MBF). Each one has its advantages and disadvantages [4,5]. T scan (Tekscan, USA) is one of the instruments very widely used. Although accurate, it is elaborate and not economical and requires training to use and analyze the measurement. Moreover, it does not measure the MVBF of the individual. Another popular instrument is the Dental Prescale System from GC, Japan. Again, it is an accurate instrument but not easy to use, requires training, is not economical, and requires a separate device to analyze the results [4,5]. Recently, a new device called “BYTE” has been developed by Innovatios Technology Bangalore, India [6,7]. The development and validation study of this novel indigenous device was presented in our earlier publication [5,6]. This paper aims to evaluate the reliability of the BYTE device through a laboratory calibration study.

## Materials and methods

The laboratory validation study was conducted at Essjay Technomeasure Private Limited, a company based in Kolkata, India. This company specializes in calibration engineering and allied services. It holds certifications from ISO 9001 and ISO 45001, indicating its commitment to quality management and occupational health and safety management systems, respectively. The study involved testing and calibration of a device known as the BYTE to know its reliability [7]. These procedures were carried out in accordance with the standards set by ISO/IEC 17025 and ISO-9001, which pertain to the competence of testing and calibration laboratories and quality management systems, respectively. The environmental conditions during the measurement process were maintained at a temperature of 25 ± 2°C and a relative humidity of 50 ± 10% RH. The calibration certificate issued for this process bears the number 2310125/I443(A)/SK/01.

**Figure 1:**
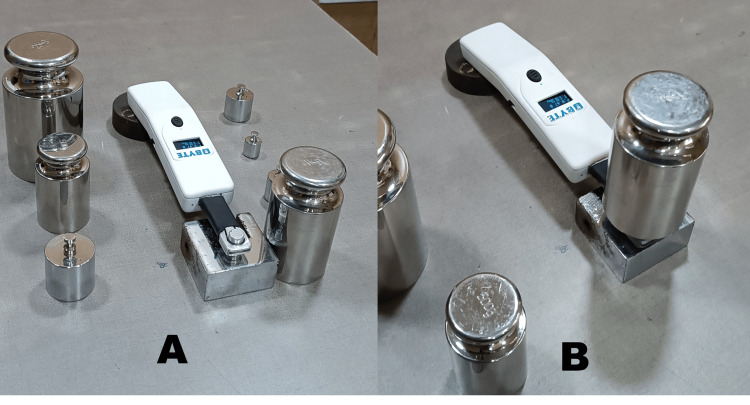
Device head supported with the jig (A) and device under calibration (B).

The BYTE device is composed of two main parts: the head and the body. The head part, which is made of stainless steel, features a circular biting portion with a diameter of approximately 10 mm. This head part houses a piezoresistive sensor that changes its resistance when pressure is applied. This change in resistance is analyzed by the firmware housed in the body part of the device. The result of this analysis, which is the recorded force in Newtons (N), is displayed on an LCD screen [5,6]. To evaluate the reliability of the BYTE device, the circular biting part of the instrument was subjected to various load cells. It is recommended that the circular biting part should be pressed uniformly for the sensor to function optimally and yield accurate and repeatable results. To ensure this uniform pressure, a custom metallic jig was fabricated to securely hold the head part of the device while loading the biting portion with various loads (Figure 1).

Standard load cells were used to apply force on the biting part as recommended. The piezoresistive sensor used in the BYTE device is sourced from Tekscan, USA [5,6]. This sensor is capable of analyzing loads up to 444 N. Therefore, standard load cells of various weights ranging from 3 N to 444 N were used sequentially at every 3 N interval. Each load was maintained for about two to three seconds, and the result was observed on the device under calibration (DUC) and noted down. This process was repeated in three sets, resulting in three measurements being taken for each standard load. The average reading was then determined from these measurements. From the average readings, an accuracy error was calculated. The data were then tabulated and subjected to statistical analysis interclass correlation coefficient for further interpretation.

## Results

The data collected during the calibration test were tabulated and subjected to statistical evaluation using IBM SPSS Statistics for Windows, version 26.0 (released 2019, IBM Corp., Armonk, NY). As per this calibration test, DUC measured load in Newtons (N) during the test. The test results are in a seven-column table: Observation No., Standard Load in N, DUC results in N Set-I, N Set-2, and N Set-3, Average Reading, and Accuracy Error. To calculate the DUC accuracy error, the standard load was subtracted from the average reading, and the absolute value was taken (Table 1).

**Table 1:** Observation No., standard load in N, DUC results in N Set-I, N Set-2, and N Set-3, average reading, and accuracy error N: Newton, DUC: device under calibration

Obs. No.	Standard load applied in N	Observed results on DUC* in N Set-I	Observed results on DUC* in N Set-2	Observed results on DUC* in N Set-3	Avg. reading	Accuracy error
1	3	2.98	2.99	2.99	2.987	0.333
2	6	5.98	5.97	5.97	5.973	0.333
3	9	9.97	9.98	9.97	9.973	0.333
4	12	11.95	11.95	11.97	11.957	0.667
5	15	14.91	14.93	14.92	14.92	0.667
6	18	17.9	17.92	17.91	17.91	0.667
7	21	20.85	20.86	20.88	20.863	1
8	24	23.87	23.85	23.86	23.86	0.667
9	27	26.84	26.83	26.85	26.84	0.667
10	30	29.81	29.81	29.83	29.817	0.667
11	33	32.79	32.8	32.81	32.8	0.667
12	36	35.74	35.75	35.77	35.753	1
13	39	38.72	38.73	38.74	38.73	0.667
14	42	41.72	41.72	41.73	41.723	0.333
15	45	44.69	44.71	44.71	44.703	0.667
16	48	47.68	47.66	47.69	47.677	1
17	51	50.65	50.67	50.67	50.663	0.667
18	54	53.58	53.56	53.54	53.56	1.333
19	57	56.54	56.52	56.53	56.53	0.667
20	60	59.51	59.5	59.48	59.497	1
21	63	62.43	62.42	62.42	62.423	0.333
22	66	65.37	65.35	65.34	65.353	1
23	69	68.34	68.32	68.31	68.323	1
24	72	71.31	71.3	71.28	71.297	1
25	75	74.28	74.29	74.24	74.27	1.667
26	78	78.25	78.26	78.22	78.243	1.333
27	81	80.23	80.21	80.21	80.217	0.667
28	84	83.17	83.15	83.14	83.153	1
29	87	86.14	86.13	86.13	86.133	0.333
30	90	89.11	89.12	89.09	89.107	1
31	93	93.09	93.08	93.06	93.077	1
32	96	96.06	96.07	96.04	96.057	1
33	99	98.03	98.05	98.05	98.043	0.667
34	102	101.01	101.04	101.03	101.027	1
35	105	103.99	103.97	103.96	103.973	1
36	108	106.95	106.92	106.91	106.927	1.333
37	111	109.92	109.94	109.95	109.937	1
38	114	112.89	112.86	112.87	112.873	1
39	117	115.85	115.83	115.81	115.83	1.333
40	120	118.82	118.81	118.78	118.803	1.333
41	123	121.8	121.81	121.78	121.797	1
42	126	124.79	124.77	124.75	124.77	1.333
43	129	127.77	127.74	127.75	127.753	1
44	132	130.76	130.75	130.74	130.75	0.667
45	135	133.74	133.72	133.71	133.723	1
46	138	136.7	136.71	136.68	136.697	1
47	141	139.67	139.65	139.64	139.653	1
48	144	142.62	142.64	142.61	142.623	1
49	147	145.59	145.57	145.55	145.57	1.333
50	150	148.53	148.54	148.56	148.543	1
51	153	151.51	151.49	151.48	151.493	1
52	156	154.5	154.51	154.47	154.493	1.333
53	159	157.47	157.49	157.49	157.483	0.667
54	162	160.56	160.54	160.55	160.55	0.667
55	165	163.47	163.45	163.48	163.467	1
56	168	166.25	166.21	166.24	166.233	1.333
57	171	169.64	169.63	169.61	169.627	1
58	174	172.41	172.38	172.37	172.387	1.333
59	177	175.58	175.55	175.56	175.563	1
60	180	178.69	178.72	178.71	178.707	1
61	183	181.45	181.47	181.51	181.477	2
62	186	184.62	184.64	184.65	184.637	1
63	189	187.77	187.79	187.76	187.773	1
64	192	190.69	190.71	190.68	190.693	1
65	195	193.66	193.67	193.64	193.657	1
66	198	196.71	196.73	196.74	196.727	1
67	201	199.56	199.58	199.53	199.557	1.667
68	204	202.52	202.56	202.55	202.543	1.333
69	207	205.51	205.53	205.55	205.53	1.333
70	210	208.47	208.52	208.49	208.493	1.667
71	213	211.43	211.46	211.45	211.447	1
72	216	214.41	214.43	214.44	214.427	1
73	219	217.39	217.43	217.41	217.41	1.333
74	222	220.34	220.32	220.3	220.32	1.333
75	225	223.42	223.44	223.46	223.44	1.333
76	228	226.41	226.38	226.39	226.393	1
77	231	229.57	229.59	229.56	229.573	1
78	234	232.52	232.54	232.53	232.53	0.667
79	237	237.34	237.37	237.36	237.357	1
80	240	238.74	238.71	238.74	238.73	1
81	243	241.69	241.68	241.67	241.68	0.667
82	246	243.54	243.56	243.52	243.54	1.333
83	249	247.45	247.42	247.47	247.447	1.667
84	252	250.39	250.36	250.34	250.363	1.667
85	255	253.34	253.31	253.32	253.323	1
86	258	256.21	256.22	256.23	256.22	0.667
87	261	259.26	259.24	259.27	259.257	1
88	264	262.21	262.24	262.23	262.227	1
89	267	265.17	265.19	265.15	265.17	1.333
90	270	268.14	268.11	268.13	268.127	1
91	273	271.11	271.13	271.1	271.113	1
92	276	274.13	274.15	274.11	274.13	1.333
93	279	277.09	277.11	277.08	277.093	1
94	282	280.07	280.09	280.05	280.07	1.333
95	285	283.04	283.04	283.02	283.033	0.667
96	288	286.05	286.03	286.01	286.03	1.333
97	291	289.04	289.01	289.02	289.023	1
98	294	291.01	291.03	290.99	291.01	1.333
99	297	294.99	294.98	294.95	294.973	1.333
100	300	297.96	297.97	297.94	297.957	1
101	303	300.92	300.93	300.91	300.92	0.667
102	306	303.91	303.88	303.86	303.883	1.667
103	309	306.95	306.9	306.92	306.923	1.667
104	312	309.92	309.91	309.88	309.903	1.333
105	315	312.89	312.87	312.88	312.88	0.667
106	318	315.88	315.86	315.85	315.863	1
107	321	318.86	318.84	318.83	318.843	1
108	324	321.84	321.82	321.85	321.837	1
109	327	324.81	324.79	324.82	324.807	1
110	330	327.77	327.75	327.74	327.753	1
111	333	330.78	330.77	330.75	330.767	1
112	336	333.82	333.84	333.81	333.823	1
113	339	336.74	336.72	336.71	336.723	1
114	342	339.72	339.71	339.69	339.707	1
115	345	342.81	342.77	342.78	342.787	1.333
116	348	345.73	345.75	345.76	345.755	0.333
117	351	348.68	348.66	348.65	348.663	1
118	354	351.65	351.64	351.62	351.637	1
119	357	354.75	354.72	354.73	354.733	1
120	360	357.69	357.66	357.68	357.677	1
121	363	360.64	360.61	360.62	360.623	1
122	366	363.62	363.59	363.61	363.607	1
123	369	366.61	366.58	366.59	366.593	1
124	372	369.57	369.55	369.54	369.553	1
125	375	372.55	372.52	372.51	372.527	1.333
126	378	375.51	375.48	375.47	375.487	1.333
127	381	379.48	379.46	379.45	379.463	1
128	384	381.44	381.43	381.41	381.427	1
129	387	384.42	384.41	384.39	384.407	1
130	390	387.43	387.39	387.38	387.4	1.667
131	393	390.37	390.36	390.33	390.353	1.333
132	396	393.41	393.38	393.36	393.383	1.667
133	399	396.38	396.34	396.35	396.357	1.333
134	402	399.35	399.32	399.31	399.327	1.333
135	405	402.32	402.31	402.34	402.323	1
136	408	405.29	405.26	405.24	405.263	1.667
137	411	408.26	408.23	408.22	408.237	1.333
138	414	411.31	411.27	411.29	411.29	1.333
139	417	414.28	414.25	414.23	414.253	1.667
140	420	417.26	417.24	417.22	417.24	1.333
141	423	420.22	420.19	420.17	420.193	1.667
142	426	423.21	423.18	423.17	423.187	1.333
143	429	426.19	426.16	426.15	426.167	1.333
144	432	429.15	429.12	429.11	429.127	1.333
145	435	432.12	432.09	432.08	432.097	1.333
146	438	435.14	435.17	435.15	435.153	1
147	441	438.09	438.06	438.05	438.067	1.333
148	444	440.06	440.05	440.04	440.05	0.667

The intraclass correlation coefficient (Table 2) shows that single and average measurements are 1.000, indicating a complete rater dependability. This means that different raters give the same subjects the same ratings. We are convinced that the true ICC is 1.000 because the 95% confidence interval is 1.000. We tested the null hypothesis that the ICC is 0, which suggests unreliable raters, with the F test with a true value of 0. The F value is large, and the p-value is tiny. Therefore, we may reject the null hypothesis and conclude that the ICC is substantially different from 0. A two-way mixed-effects model treats raters as fixed effects and participants as random effects. This suggests that we are interested in the reliability of the raters we chose for the study, not in generalizing to other raters with similar qualities. 

**Table 2:** Interclass correlation coefficient F test: Fisher's test, df: degree of freedom, Sig: significance (p < 0.05)

Intraclass correlation coefficient								
	Intraclass correlation^b^	95% confidence interval		F test with true value 0				
		Lower bound	Upper bound	Value	df1	df2	Sig	
Single measures	1.000^a^	1.000	1.000	196692581.132	147	294	.000	
Average measures	1.000^c^	1.000	1.000	196692581.132	147	294	.000	
Two-way mixed-effects model where people effects are random and measures effects are fixed.								
a. The estimator is the same, whether the interaction effect is present or not.								
b. Type A intraclass correlation coefficients using an absolute agreement definition.								
c. This estimate is computed assuming the interaction effect is absent because it is not estimable otherwise.								

As per this calibration test, the DUC's maximum accuracy error is 1.667 N at 75 N, according to test data. At numerous standard loads, the minimum accuracy error is 0.333 N. The DUC marginally underestimates load with an average accuracy error of 0.833 N. Test findings reveal that the DUC performs consistently throughout three sets of measurements, as the results are extremely similar.

## Discussion

Regardless of the state of the occlusal condition, the bite force plays a vital role in masticatory performance [8,9]. Measuring the biting force is considered a critical step in diagnostic and treatment planning in restorative dentistry [10-12]. Various devices have been used in the literature to measure bite force having their own merits and demerits [4]. The basic requirement of a bite force measuring device is that it should be accurate, consistent, economical, and simple to use. The “BYTE” device was proposed as an economical, easy-to-use, and reliable instrument to measure the maximum bite force [7]. It has a flexiforce sensor (Tekscan, USA) encased in the two plates of stainless steel in the head part. Moreover, all the hardware in the body part to analyze the change in resistance due to applied force, made in polypropylene plastic. The circular biting portion of the head part is kept on the occlusal surface of the tooth, and the patient is asked to bite over it to measure the bite force. A protective silicone cap is advised to be put on the biting portion during recording. It is reported to be portable, wireless, and easy to disinfect [5,6]. A patent has been granted by the government of India (patent number 489519).

This study evaluates the validity of the instrument by a mechanical calibration test. Calibration tests verify the accuracy and dependability of measuring instruments, tools, and devices. A calibration test compares the DUC output to a more accurate reference standard. A physical measurement device or test data can be the reference standard. The calibration test can show the DUC's divergence from the reference standard and assist in rectifying it. A calibration test can also confirm that the DUC satisfies its use standards.

As per this calibration test report, the device's maximum accuracy error is 1.667 N at 75 N. At numerous standard loads, the minimum accuracy error is 0.333 N. The device marginally underestimates load with an average accuracy error of 0.833 N. The results are found within permissible limits at ±10% to 15% on MSD. Test findings reveal that the device performs consistently throughout three sets of measurements. A bite force measuring instrument with a similar type of sensor was reported by Testa et al. in 2016 [13]. They reported that the sensitivity of the device was rather increased due to the housing compared to the bare instrument. In our study, calibration tests were done without the housing, and the device was performed consistently. A miniature bite force recorder was presented by Singh et al. in 2011 [14]. It was a strain gauge base metallic bite fork that was calibrated using a universal testing machine with a force range between 10 kg and 85 kg. In our study, standard loads starting from 3 N to 444 N were applied. Measurements were taken at every 3 N interval thrice, and the mean was taken for each load. A similar type of bite force recorder was presented by Waltimo A et al. in 1993 [15]. It has a quartz force transducer in a metallic housing covered by rubber. The calibration test of the instrument was done by compression test machine. The instrument was tested with various loads to assess reliability. For each load, 10 recordings were made to calculate the mean value and standard deviation. They concluded that the housing had no bearing on the sensor's linearity. After roughly 500 clinical measures, the housing was tested once again using the same compression test apparatus to confirm the validity of the methodology. A prototype loadpad pressure mapping sensor, of the capacity type, was shown by Steffen C et al. in 2023 [16]. They claimed it to be effective and reliable in edentulous patients for measuring bite force and also in segmental mandibular resection patients. They again used a universal testing machine for validation and calibration tests. Two approaches were used one continuous loading and the other cyclic loading. It was also assessed how the silicone covering affected the recording of bite force and the reliability of the device. In our study, various standard weights starting from 3 N to 444 N at every 3 N interval were loaded onto the biting part of the device without any silicone or other protective layer to analyze the validity and reliability.

This study does have a few limitations. First, the reliability of the device was tested in a controlled laboratory setting, which may not accurately reflect the conditions within the mouth. The most crucial aspect of the test is the application of uniform pressure to the biting portion of the device. To achieve this in patients, it is recommended to use a resin stent during the recording of bite force. In addition, this study did not evaluate the impact of a protective silicone cap or cover. Furthermore, the effects of different loading conditions, such as continuous and cyclic, need to be examined.

## Conclusions

In the course of our laboratory validation test, we discovered that the BYTE device demonstrated a high degree of reliability and consistency. The maximum accuracy error was observed to fluctuate within a range of 0.333 N to 1.667 N. This variation was well within the acceptable limits, indicating a high level of precision.

Furthermore, the instrument’s accuracy errors were found to be minor and remained consistent across different load levels. This consistency is a testament to the device’s steady performance under varying conditions. Given these findings, it is our recommendation that the BYTE device is highly suitable for use in both clinical and experimental settings, particularly for the measurement of bite force.
